# The outcome of prostate cancer patients treated with curative intent strongly depends on survival after metastatic progression

**DOI:** 10.1186/s12885-017-3617-6

**Published:** 2017-09-18

**Authors:** Mariarosa Pascale, Che Ngwa Azinwi, Barbara Marongiu, Gianfranco Pesce, Flavio Stoffel, Enrico Roggero

**Affiliations:** 1 Medical oncology Unit, Oncology Institute of Southern Switzerland (IOSI), 6500 Bellinzona, Switzerland; 2 Radiation oncology unit, Oncology Institute of Southern Switzerland (IOSI), 6500 Bellinzona / 6900 Lugano, Switzerland; 3 Urology unit, Ospedale San Giovanni, 6500 Bellinzona, Switzerland

**Keywords:** Prostate cancer, Metastasis, Prognosis, Curative intent, Radiotherapy, Radical prostatectomy

## Abstract

**Background:**

Five-year survival in patients with localized prostate cancer (PCa) is nearly 100%, but metastatic disease still remains incurable. Clinical management of metastatic patients has become increasingly complex as novel therapeutic strategies have emerged. This study aims at evaluating the impact of the first metastatic progression on the outcome of PCa patients treated with curative intent.

**Methods:**

The analysis was conducted using data of 913 cases of localized PCa diagnosed between 2000 and 2014. All patients were treated with curative surgery (*N* = 382) or radiotherapy (*N* = 531) with or without adjuvant therapy. All metastases were radiologically documented. The prognostic impact of the first site of metastasis on metastasis-free survival (MFS) and PCa-specific survival (PCaSS) was investigated by univariate and multivariate analyses.

**Results:**

One hundred and thirty-six (14.9%) patients developed a metastatic hormone-sensitive PCa and had a median PCaSS of 50.4 months after first metastatic progression. Bone (*N* = 50, 36.8%) and LN or locoregional (*N* = 52, 38.2%) metastases occurred more frequently with a median PCaSS of 39.7 and 137 months respectively (*p* < 0.0001). Seven patients developed visceral metastasis only (5.1%; liver, lung, brain) and 27 (19.9%) concurrent metastases; this last group was associated with the worst survival with a median value of only 17 months. Thus, each subgroup exhibited a survival after metastasis significantly different from each other. In multivariate analysis the site of the first metastasis was an independent prognostic factor for PCaSS along with Gleason score at diagnosis. The correlation between survival and first site of metastasis was confirmed separately for each therapy subgroup. Median metastasis-free survival from primary diagnosis to first metastasis was not correlated with the first site of metastasis.

**Conclusions:**

In non-metastatic PCa patients treated with curative intent, the PCa-specific survival time depends on the time after metastatic progression rather than the time from diagnosis to metastasis. Moreover, the site of first metastasis is an independent prognostic factor for PCaSS. Our data confirm that the first metastatic event may confer a differential prognostic impact and may help in identifying patient at high risk of death supporting the treatment-decision making process following metastatic progression.

**Electronic supplementary material:**

The online version of this article (10.1186/s12885-017-3617-6) contains supplementary material, which is available to authorized users.

## Background

Prostate cancer (PCa) is the most frequent tumor in men and a major leading cause of cancer-related deaths in developed countries [1]. Many men have tumors that grow very slowly, whereas others develop very aggressive disease, which metastasizes rapidly spreading to elsewhere in the body. The majority of patients with localized PCa will be cured after local therapy with five-year survival near 100% [2]; but once the tumor progresses developing distant metastasis, the disease often become incurable [2, 3]. Indeed advanced prostate cancer still accounts for the majority of the mortality from this disease [4, 5], although survival might be extensive [6]. The most common metastatic sites are bone and lymph nodes (LN) [4, 7–14], but visceral metastases may also be present [4, 5, 7, 8, 10–14] and may be associated with a more severe clinical course [5, 8, 12, 13, 15–17].

In the last years several studies have suggested the prognostic importance of the site of metastasis in men with de novo metastatic PCa [8, 11] or metastatic castration-resistant prostate cancer (mCRPC) [5, 13]. To our knowledge, few studies [9, 12, 14, 18] have assessed the impact of location of metastatic disease on the outcome of men with PCa after receiving curative treatment. Shao et al. [18] firstly demonstrated that primary treatment may make a difference with regard to survival time after metastasis; both Nini et al. [12] and Moschini et al. [14] found that nodal and local recurrence have a more favorable prognosis compared with skeletal and visceral metastases in pN+ patients treated with radical prostatectomy. Local and nodal site were the most frequent primary location of metastasis in patients treated with both radiotherapy [9] and prostatectomy [12, 14].

In this work we sought to address this issue by using a cohort of non-metastatic primary PCa patients who underwent prostatectomy or radiotherapy with curative intent with the aim of evaluating the impact of the first metastatic event on PCa-specific survival in order to respond to the need to improve the treatment-decision making process following metastatic progression.

## Methods

### Patients

An observational analysis was performed by using a database of 1364 patients diagnosed with PCa from 2000 to 2014 at Oncology Institute of Southern Switzerland (IOSI) and Urology Unit of San Giovanni Hospital (OSG). Clinical, pathological, and demographic data were registered. Agreement was obtained from the Ethics Committee of Canton Ticino to collect and analyze data without disclosing patient identifiers. Follow-up data were collected through August 2015.

**Fig. 1:**
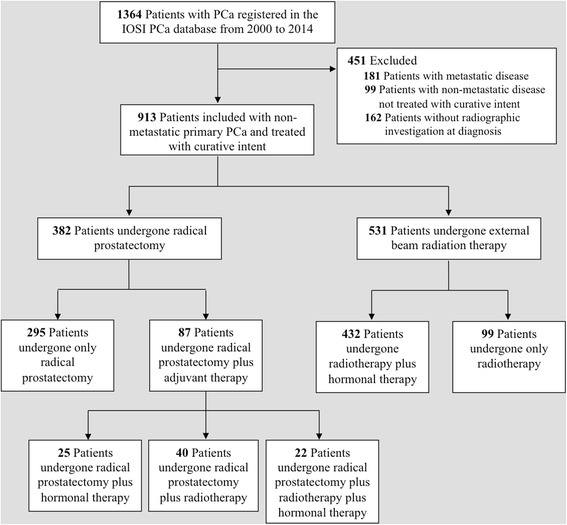
Cohort selection for non-metastatic primary PCa patients treated with curative intent

Radical prostatectomy (*N* = 382) and external beam radiation therapy (*N* = 531) with curative intent were considered. Some patients received adjuvant therapy after prostatectomy (*N* = 87; 22.8%), including hormonal therapy (*N* = 25) or radiation therapy (*N* = 40) or both (*N* = 22). Most of patients treated with radiotherapy received a concomitant hormonal treatment (*N* = 432; 81.3%) (Fig. 1). First-line therapy was chosen according to standard clinical practice.

### Definitions of metastasis subgroups

Patients developing a metastatic hormone-sensitive disease, defined as a tumor responding to hormone therapy, were categorized into one of the following subgroups according to the site of the first metastatic progression of the disease after curative treatment: 1) presence of exclusive bone metastasis (Bone-only subgroup); 2) presence of LN or locoregional metastasis, alone or concomitant (Locoregional/LN-only subgroup); 3) presence of exclusive visceral disease (Visceral-only subgroup) and 4) presence of multiple sites of metastasis (Multiple site subgroup). Visceral disease was defined as metastatic disease to liver, lung, brain and other organ sites. Patients with multiple metastatic sites were further stratified in one of the following categories: a) patients with bone metastasis with LN or locoregional disease and b) patients with visceral disease with bone, nodal or locoregional involvement. All metastases were radiologically documented (MRI or CT scan or Choline-PET scan or bone scintigraphy). Follow-up visits and imaging examinations were performed according to standard clinical practice or in case of symptomatic disease.

### Outcomes

Study endpoints were: PCa-specific survival (PCaSS), defined as the time interval from the date of primary PCa diagnosis to the date of PCa related-death or last follow-up; metastasis-free survival (MFS), defined as the time interval from the date of primary PCa diagnosis to the first radiographic metastasis; PCa-specific survival after metastasis (PCaSS after metastasis), defined as the time interval from the date of the first radiographic metastasis to the date of PCa related-death or last follow-up.

### Statistical analysis

Demographic characteristics of patients were reported using median and interquartile ranges for continuous variables and frequencies and proportions for categorical variables. The independent t test and the chi-square test were used to assess associations between continuous and categorical variables, respectively.

Estimates of medians, rate and 95% confidence intervals (CIs) were determined using the Kaplan–Meier method. Patients were censored if they were still alive or they were lost to follow-up. Differences in survival times were evaluated using the log-rank test. A multivariable Cox regression analysis was used to assess the prognostic impact of the first site of metastasis on PCaSS and MFS after adjusting for other covariates that might partially influence the outcome. All variables associated with univariate value of *p* ≤ 0.05 were included in the multivariate model.

All tests were considered statistically significant at *p* ≤ 0.05. Statistical analyses were carried out using software STATA software (StataCorp. 2011. Stata Statistical Software: Release 12. College Station, TX: StataCorp LP).

## Results

### Patients

Of the 1364 men with PCa registered in our database, 913 patients with localized disease who underwent curative treatment were identified. Histologically all of them were acinar adenocarcinoma of the prostate. In particular, 382 patients underwent radical prostatectomy, without (*N* = 295) or with adjuvant radiation (*N* = 40) or hormonal (*N* = 25) therapy or both (*N* = 22), and 531 men were treated with external beam radiation treatment (*N* = 531), without (*N* = 99) or with hormonal therapy (*N* = 432). Median age at diagnosis of the entire cohort was 67 years (IQR 62.7–71.8). After a median follow-up of 5.7 years (IQR 2.9–8.8) from the date of primary tumor diagnosis, 60 patients (6.6%) have died. Five-year PCaSS rate was 97.2% (95% CI 95.6–98.2). Of 913 patients, 136 (14.9%) developed a metastatic hormone-sensitive PCa.

Disease characteristics of the cohort according to the development of the metastatic disease and curative treatment are summarized in Tables 1 and 2, respectively. Patients who progressed to metastasis had a lower age at diagnosis (median value 65.3 vs 67.2 years old), higher PSA, elevated Gleason Score and more LN involvement than patients who did not progress (Table 1). As expected, patients treated with prostatectomy were younger (median value 63.4 vs 70.3 years old) and had different disease characteristics compared with patients who underwent radiotherapy (Table 2). But no significant statistically differences between the two treatment modalities were found according to the site of metastasis at first progression of the disease after curative treatment (*p* = 0.158).

**Table 1:** Clinical-pathological characteristics of 913 primary PCa patients by first metastatic progression

Parameter at diagnosis	No recist progression N (%)	First recist progression N (%)	*P*-value
Age (years)			
< 50	6 (0.8)	2 (1.5)	0.036*
≥ 50 and <60	106 (13.6)	29 (21.3)	
≥ 60 and <75	570 (73.4)	97 (71.3)	
≥ 75 and <80	80 (10.3)	8 (5.9)	
≥ 80	15 (1.9)	0	
Stage			
T1	175 (22.6)	12 (9)	< 0.001*
T2	337 (43.5)	27 (20.1)	
T3	254 (32.8)	88 (65.7)	
T4	9 (1.1)	7 (5.2)	
*Missing*	*2*	*2*	
*Lymph nodes*			
Negative	725 (94.4)	111 (83.5)	< 0.001*
Positive	43 (5.6)	22 (16.5)	
Missing	9	3	
PSA (ng/ml)			
< 10	329 (43.6)	40 (38.5)	0.001*
≥ 10 and <20	282 (37.3)	28 (26.9)	
> 20	144 (19.1)	36 (34.6)	
Missing	22	32	
Gleason Score			
3–6	238 (32)	18 (16.8)	< 0.001*
7	326 (43.8)	37 (34.6)	
8–10	180 (24.2)	52 (48.6)	
Missing	33	29	
Total (N)	777	136	

*Significant value

**Table 2:** Clinical-pathological characteristics of 913 primary PCa patients by curative intent treatment

First line therapy	S/S + other N (%)	RT/RT + HO N (%)	*P*-value
Age (years)			
< 50	6 (1.6)	2 (0.4)	< 0.001*
≥ 50 and <60	107 (28)	28 (5.3)	
≥ 60 and <75	266 (69.6)	401 (75.5)	
≥ 75 and <80	2 (0.5)	86 (16.2)	
≥ 80	1 (0.3)	14 (2.6)	
Stage			
T1-T2	191 (50.1)	360 (68.2)	< 0.001*
T3	186 (48.8)	156 (29.5)	
T4	4 (1.1)	12 (2.3)	
Missing	1	3	
Lymph nodes			
N0	322 (85.2)	514 (98.3)	< 0.001*
N1	56 (14.8)	9 (1.7)	
Missing	4	8	
PSA (ng/ml)			
< 10	189 (56.4)	180 (34.3)	< 0.001*
10–20	101 (30.2)	209 (39.9)	
≥ 20	45 (13.4)	135 (25.8)	
Missing	47	7	
Gleason Score			
3–6	79 (22.1)	177 (35.9)	< 0.001*
7	192 (53.6)	171 (34.7)	
8–10	87 (24.3)	145 (29.4)	
Missing	24	38	
First metastatic event			
Bone-only	30 (40.54)	20 (32.26)	0.158
Loco/LN-only	28 (37.4)	24 (38.71)	
Visceral-only	1 (1.35)	6 (9.68)	
Multiple site	15 (20.27)	12 (19.35)	
Total (N)	382	531	

*S* Surgery, *RT* Radiotherapy, *HO* Hormonal therapy; other: adjuvant therapy. *Significant value

### Metastases at first progression

#### Distribution of metastatic sites at first progression

At the time of first progression, metastases were most often to LN and/or locoregional area (*N* = 52, 38.2%) and to bone (*N* = 50, 36.8%). The remaining patients developed visceral-only disease (*N* = 7, 5.1%) or metastases involving multiple sites (*N* = 27, 19.8%; of which *N* = 16, 11.8% bone metastasis with LN or locoregional disease and *N* = 11, 8.1% visceral disease with bone, nodal or locoregional involvement). Visceral disease involved liver (*N* = 3), lung (*N* = 11), liver and lung (*N* = 2), liver and brain (*N* = 1), other (*N* = 1). Therapy after diagnosis of metastasis is reported in Additional file 1: Table S1.

#### PCa-specific survival

Five-year PCaSS rate were 100% and 85% (95% CI 77.5–90.2) for patients who did not develop and who developed metastasis, respectively (Additional file 2: Figure S1). When patients were stratified according to the first site of metastasis, the Locoregional/LN-only subgroup had significantly higher 5-year PCaSS survival rate compared with the Bone-only and the Multiple site subgroups (96.1%, 95%CI: 85.2–99.0; 84.2%, 95%CI: 69.7–92.2; 64.0%, 95%CI: 42.0–79.5 respectively; *p* = 0.0126) (Additional file 3: Figure S2).

#### Metastasis-free survival

**Fig. 2:**
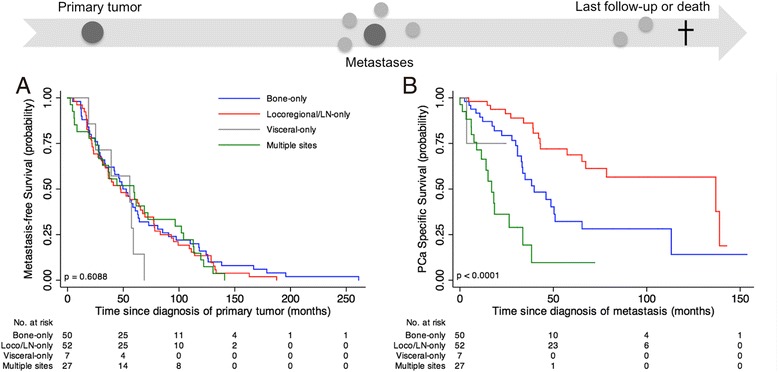
Survival curves by first metastatic event of 136 PCa patients progressing after curative treatment of primary tumor. **a** Metastasis-free survival curves (**b**) PCa-specific survival curves. *p*-value from log-rank test is reported. Numbers of at risk (still alive) patients are indicated below the x-axis

Median MFS was 49.6 months (95% CI 39.1–60.1) with estimated 5- and 10-year MFS of 41.9% (95% CI 33.6–50.0) and 13.2% (95% CI 8.2–19.5), respectively. No statistically significant differences were revealed in MFS according to metastasis subgroups (Fig. 2a; *p* = 0.6088). MFS was also analyzed according to curative therapy subgroups and neither radical prostatectomy nor radiotherapy showed statistically significant differences in MFS according to metastasis subgroups (Additional file 4: Figure S3; *p* = 0.4649 for radical prostatectomy and *p* = 0.8222 for radiotherapy). Multivariable Cox regression analysis for MFS was not statistically significant (*p* = 0.4795).

#### PCa-specific survival after first metastasis

##### 3.2.4.1.Overall analysis

Median PCaSS after metastasis was 50.4 months (95% CI 39.1–78.5). After stratifying patients according to the first metastatic site, median PCaSS after metastasis were 39.7 months (95% CI 32.9–51) for the Bone-only subgroup, 137 months (95% CI 65.3–nr) for the Locoregional/LN-only subgroup and 17 months (95% CI 8.9–33.8) for the Multiple site subgroup (*p* < 0.001) (Fig. 2b). For this last subgroup, the presence of visceral metastases worsened the prognosis by 3 months (18.1 vs 15.1). Median PCaSS after metastasis for the Visceral-only subgroup was not reached.

##### 3.2.4.2.Analysis by therapy groups

**Fig. 3:**
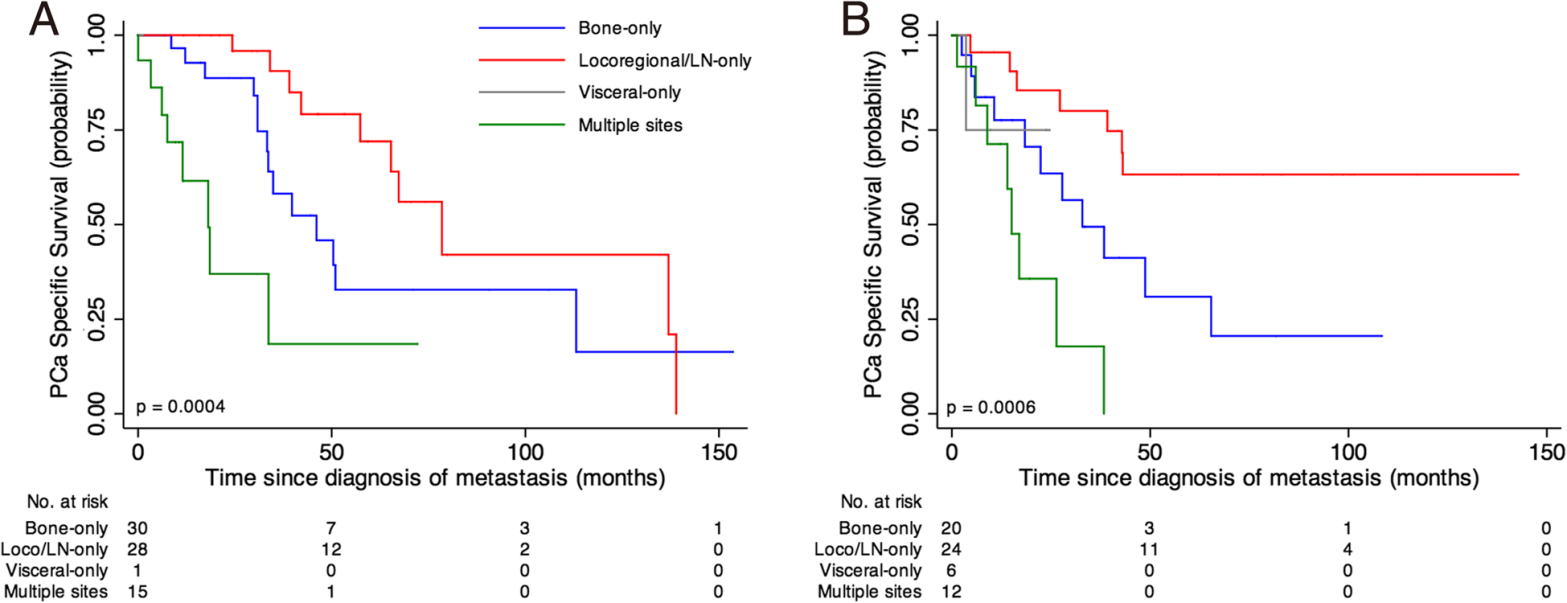
PCa-specific survival curves by first metastatic event of 136 PCa patients progressing after curative treatment of primary tumor. **a** Radical prostatectomy subgroup. **b** Radiotherapy subgroup. *p*-value from log-rank test is reported. Numbers of at risk (still alive) patients are indicated below the x-axis

To further investigate the impact of the site of the metastatic disease on the outcome, additional analysis was performed after stratifying patients according to the therapy subgroup. Overall median PCaSS after metastasis were 57.4 months (95% CI 39.1–113.1) and 42.9 months (95% CI 27.2–nr) for patients treated with surgery and radiation therapy, respectively (*p* = 0.5928). In the prostatectomy subgroup, median PCaSS after metastasis were 46.1 months (95% CI 33.3–113.1) for men with bone-only metastases, 78.5 months (95% CI 57.4–nr) for men with Locoregional/LN-only metastases and 18.1 months (95% CI 6.1–nr) for men with multiple locations of the metastastic disease (*p* = 0.0004; Fig. 3a). Whereas PCaSS after metastasis in the radiotherapy subgroup were 32.9 months (95% CI 18.4–65.4) and 15.07 (95% CI 6–nr) for men with bone-only metastases and multiple metastatic sites, respectively; men with Locoregional/LN metastasis did not reach median survival (*p* = 0.0006) (Fig. 3b). None of subgroups of metastasis showed statistically significant differences between the two curative treatments (Logrank test, *p* = 0.2054 for Bone-only, *p* = 0.6158 for Locoregional/LN-only, *p* = 0.6321 for Multiple site).

### Multivariate analysis predicting PCa-specific survival

Multivariable Cox regression analysis showed that the first site of metastasis is an independent predictor of PCaSS (Table 3). Particularly, patients who progressed to bone-only and to multiple sites had a higher risk of dying from PCa compared with those developing Locoregional/LN-only disease with HR of 3.88 (*p* = 0.011) and 3.56, respectively (*p* = 0.019). Moreover, Gleason score was the only primary tumor parameter that represented an independent predictor of PCaSS. Patients with Gleason score 8–10 had a worst PCaSS compared with those having lower grade cancer (HR 2.45, *p* = 0.020).

**Table 3:** Multivariate analysis of prognostic factors based on Cox proportional hazards regression model in 136 primary PCa patients progressing to metastatic hormone-sensitive disease after curative treatment

Variable	Hazard ratio	95% CI	*p*-value
Age at diagnosis (years)	1.05	0.97–1.13	0.213
PSA (ng/ml)			
10–20 vs < 10	0.31	0.09–1.13	0.076
> 20 vs < 10	1.47	0.66–3.29	0.348
Stage			
T3-T4 vs T1-T2	0.82	0.31–2.38	0.687
Gleason Score			
> 7 vs ≤ 7	2.47	1.15–5.29	0.020*
Site of metastasis at first progression			
Bone-only vs Loco/LN-only	3.88	1.37–10.98	0.011*
Visceral-only vs Loco/LN-only	1.89	0.20–18.10	0.582
Multiple site vs Loco/LN-only	3.56	1.24–10.24	0.019*

*CI* Confidence interval; * significant value

## Discussion

In this data set analysis of men receiving curative radiotherapy or surgery for primary PCa, the site of the first metastasis was an independent prognostic factor for PCa death. In the entire cohort, men initially developing LN or locoregional metastases, which were the most frequent along with bone in our series as already reported by other authors [4, 9, 12, 14], had the best survival followed by those with osseous metastases. Men having metastases at multiple locations exhibited the worst prognosis; moreover, a shorter survival was associated with disease involving visceral sites too. These data are consistent with other authors who showed that multiple recurrences had a poorer prognosis than a single recurrence in pN+ patients treated with radical prostatectomy [14] and a worse outcome in presence of visceral involvement in newly metastatic patients [8, 10]. This trend in PCaSS was confirmed when the analysis was done separately for curative surgery or radiotherapy. The Locoregional/LN-only subgroup had the best prognosis in both therapy subgroups confirming previous findings in pN+ patients treated with radical prostatectomy [12, 14]. Therefore, our study highlights that PCa-specific survival strongly depends on the time after metastasis. In fact, median survival from primary diagnosis to initial metastasis (MFS) was independent of the site of the first metastatic event; indeed, each metastatic subgroup exhibited a very similar MFS with a median value of 49.6 months, very alike to that found by Ost et al. [9] in patients treated with radiotherapy. This result was confirmed also for each therapy subgroup, separately.

Our findings could be explained by the expression of different biologic characteristics that underlie the spread of PCa cells to metastatic sites. Indeed, it seems that tumor cells that spread only to LNs may have acquired specific phenotypic modifications that predispose to the preferential invasion of lymphatic vessels and access to lymph nodes [10, 12, 20, 19]. These cells might acquire the ability to spread from lymph nodes to distant organs via blood or lymphatic channels only after subsequent neoplastic transformations [3, 12, 20, 21]. Thus, PCa cells that disseminate to nodes harbor a less aggressive phenotype compared with those spreading systemically to other sites. This may explain the more favorable prognosis of patients with local/nodal recurrence compared with those with systemic disease, as already suggested by others [12]. On the other hand, visceral disease seems to be a very adverse prognostic factor, especially in de novo metastatic PCa [8] and mCRPC patients [5, 13, 15, 17]. However, it was reported as having a favorable outcome in the absence of extensive bone metastases [16] and in metastatic patients at initial diagnosis [10, 11, 22]. Interestingly, Pouessel et al. [22] analyzing patients with localized or locally advanced disease at diagnosis found that median overall survival was 6 months for patients who had a late diagnosis of liver metastasis and 14 months for whom liver was part of the initial pattern of metastases. This finding was consistent with Wang et al. [23] that showed that the outcome of liver metastasis was worse for patients whose liver metastasis was synchronous at primary PCa diagnosis than in those for whom the liver was a site of progressive PCa. In our series men having visceral-only disease were under-represented and it was impossible to make a definitive conclusion on the outcome of those patients; however, we found that the presence of visceral disease worsened the outcome of men having other metastases, as previously shown by others [8, 10]. Of note, Gleason score was the only baseline parameter predicting PCaSS endorsing its prognostic value for disease-specific survival [24] and further reinforcing that poorly differentiated cancers tend to have a more aggressive biological behavior, including high risk of metastasis [25].

Finally, the prevalence of metastasis to specific sites, mostly to bone and nodal/locoregional area [4, 9, 12, 14], has been explained biologically by the interaction between metastatic tumor cells and the organ microenvironment [26], favored by a preferential homing [27], or purely by the anatomy of vascular and lymphatic drainage from the site of the primary tumor [28].

Our work is certainly limited by its observational design and institutional registry-based study; thus, non-standardized timing for imaging, changes in treatment indication and imaging over years, inherent biases in the institution and other confounders may have been affected the results. On the other hand, being a single-center study it has allowed us to analyze a homogeneous curative cohort of non-metastatic PCa managed by standard local protocol and to demonstrate the prognostic role of the location of the metastatic hormone-sensitive disease independently in both intervention groups. Moreover, our data clarify that the time after metastatic progression rather than the time from diagnosis to metastasis strongly impacts on patients’ outcome and above all that the first metastatic event is an important factor in defining the prognosis of patients treated with curative intent and it will help in the treatment-decision making process following metastatic progression. Further studies addressing this topic are imperative to find additional parameter to stratify men with PCa into prognostic groups according to their metastatic disease. Moreover, studies investigating the biologic mechanisms underlying metastatic spread to specific locations are also needed. All these data might have important implications for the development of novel therapeutic approaches targeting the specific metastatic site that will help in selecting the optimal therapy for individual patients according to the metastatic disease they will experience.

## Conclusions

In PCa patients initially treated with curative intent, the PCa-specific survival strongly depends on the time after metastatic progression and the first location of metastasis. Thus, metastatic site may confer a differential prognostic impact and may be used to identify patients at the highest risk of death. Our results provide the conceptual framework for treating patients according to the metastatic disease and advance arguments to introduce location of metastasis as a research parameter in PCa studies.

## Additional files

Additional file 1:Table S1. Therapy after diagnosis of the first metastasis. Summary of therapies that patients received after the diagnosis of a metastasis. (DOCX 35 kb)

Additional file 2: Figure S1. PCa-specific survival curves. **(A)** Entire cohort of 913 non-metastatic primary PCa patients treated with curative intent. **(B)** Survival curves by first metastatic event. *p*-value from log-rank test is reported. Numbers of at risk (still alive) patients are indicated below the x-axis. (TIF 5180 kb)

Additional file 3: Figure S2. PCa-specific survival curves by first metastatic event of 136 PCa patients progressing after curative treatment of primary tumor. *p*-value from log-rank test is reported. Numbers of at risk (still alive) patients are indicated below the x-axis. (TIF 5704 kb)

Additional file 4: Figure S3. Metastasis-free survival curves by first metastatic event of 136 PCa patients progressing after curative treatment of primary tumor. **(A)** Radical prostatectomy subgroup. **(B)** Radiotherapy subgroup. *p*-value from log-rank test is reported. Numbers of at risk (still alive) patients are indicated below the x-axis. (TIF 5390 kb)

## Abbreviations

CI: Confidence Interval
GS: Gleason score
IQR: Interquartile range
LN: Lymph node
mCRCP: Metastatic castration-resistant prostate cancer
MFS: Metastasis-free Survival
PCa: Prostate Cancer
PCaSS: PCa-specific Survival
PSA: Prostatic Specific Antigen
