# Supplementary material for: The outcome of prostate cancer patients treated with curative intent strongly depends on survival after metastatic progression
Source: BMC Cancer. 2017 Sep 18;17:651. doi: 10.1186/s12885-017-3617-6 (PMC5604496; doi:10.1186/s12885-017-3617-6)
Supplement: Supplementary file 1 — Therapy after diagnosis of the first metastasis. Summary of therapies that patients received after the diagnosis of a metastasis. (DOCX 35 kb) [file 12885_2017_3617_MOESM1_ESM.docx]

**Supplementary table 1.** Therapy after diagnosis of the first metastasis.

| **Therapy after diagnosis of the first metastasis** | **N** |
| --- | --- |
| Hormonal therapy  Pelvic salvage radiotherapy + hormonal therapy  Chemotherapy + hormonal therapy  Palliative radiotherapy on metastasis + hormonal therapy  Palliative radiotherapy on metastasis + chemotherapy + hormonal therapy  No therapy | 61  24  14  24  4  9 |
| **Total (N)** | **136** |

*Significant value.
