# Supplementary figures and images for: The outcome of prostate cancer patients treated with curative intent strongly depends on survival after metastatic progression
Source: BMC Cancer. 2017 Sep 18;17:651. doi: 10.1186/s12885-017-3617-6 (PMC5604496; doi:10.1186/s12885-017-3617-6)

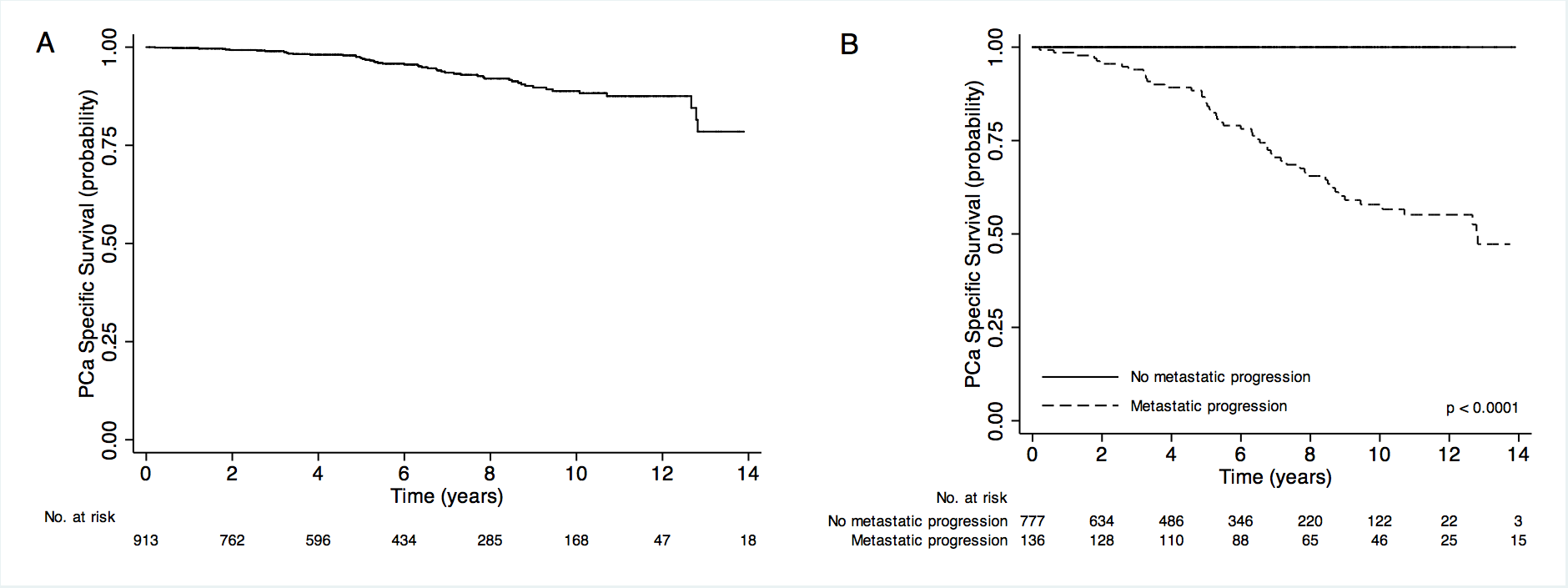

Supplement: Supplementary file 2 — PCa-specific survival curves. (A) Entire cohort of 913 non-metastatic primary PCa patients treated with curative intent. (B) Survival curves by first metastatic event. p-value from log-rank test is reported. Numbers of at risk (still alive) patients are indicated below the x-axis. (TIF 5180 kb) [file 12885_2017_3617_MOESM2_ESM.tif]

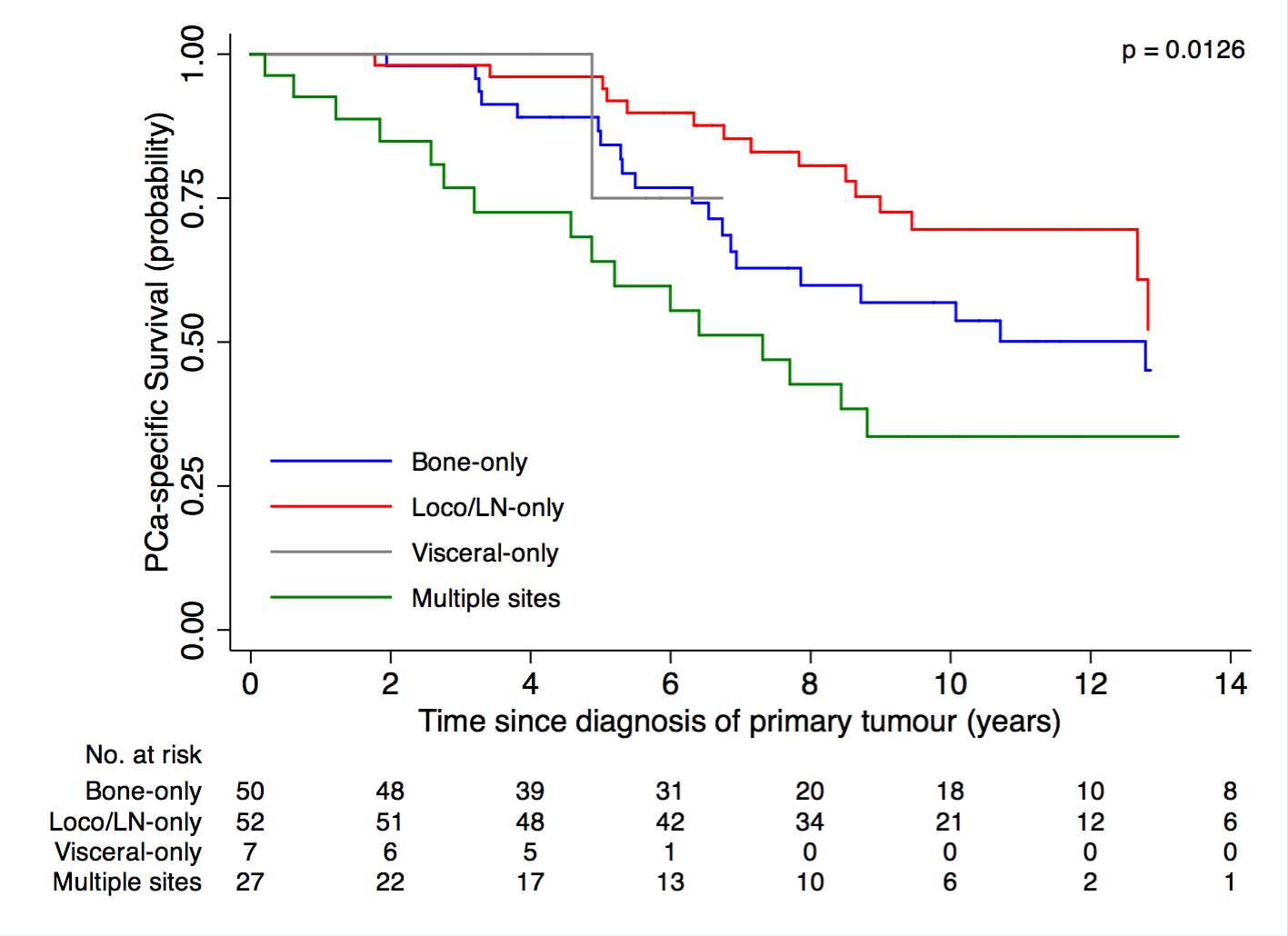

Supplement: Supplementary file 3 — PCa-specific survival curves by first metastatic event of 136 PCa patients progressing after curative treatment of primary tumor. p-value from log-rank test is reported. Numbers of at risk (still alive) patients are indicated below the x-axis. (TIF 5704 kb) [file 12885_2017_3617_MOESM3_ESM.tif]

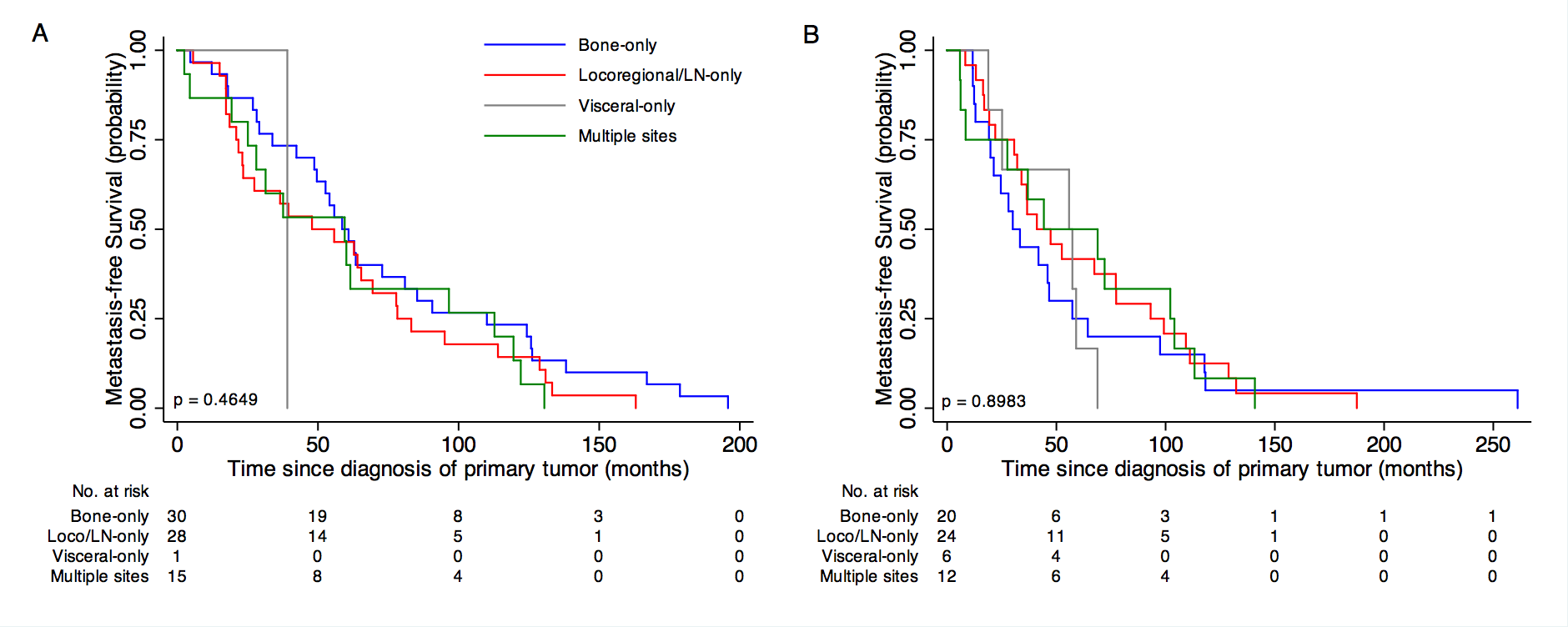

Supplement: Supplementary file 4 — Metastasis-free survival curves by first metastatic event of 136 PCa patients progressing after curative treatment of primary tumor. (A) Radical prostatectomy subgroup. (B) Radiotherapy subgroup. p-value from log-rank test is reported. Numbers of at risk (still alive) patients are indicated below the x-axis. (TIF 5390 kb) [file 12885_2017_3617_MOESM4_ESM.tif]
